# Characterization of nickel levels considering seasonal and intra-individual variation using three biological matrices

**DOI:** 10.1007/s11356-023-31252-7

**Published:** 2023-12-08

**Authors:** Jae-Hee Min, Seungho Lee, Hyoun-Ju Lim, Min-Kyung Kang, Hyunjin Son, Byoung-Gwon Kim, Young-Seoub Hong

**Affiliations:** 1 Department of Preventive Medicine, College of Medicine, Dong-A University, 32 Daesingongwon-ro, Seo-gu, Busan, 49201 Korea; 2 Environmental Health Center for Busan, Dong-A University, Busan, Korea

**Keywords:** Intra-class correlation, matrix, Nickel, Reference value, Variability

## Abstract

Nickel compounds are classified as group 1 carcinogens by the International Agency for Research on Cancer. However, only a few exposure assessment studies have been conducted on such compounds to date. In this study, we investigated the distribution of nickel in three biological types of samples (blood, serum, and urine) and its temporal variability through repeated measurements. From 2020 to 2021, blood and urine samples were collected for four times from 50 healthy participants. Nickel concentrations were determined using inductively coupled plasma mass spectrometry, and inter-individual correlation was calculated from linear mixed model. The overall geometric mean of nickel was 1.028 μg/L in blood, 0.687 μg/L in serum, and 1.464 μg/L in urine. Blood nickel was the highest in November (blood: 1.197 μg/L), and the geometric mean of nickel concentrations in the serum and urine were the highest in March (serum: 1.146 μg/L; urine: 1.893 μg/L). This matched seasonal trends for fine particulate matter concentrations from 2020 to 2021. Thus, seasonal effects significantly affect nickel levels in blood, serum, and urine. The inter-individual correlations were low as 0.081 for blood and 0.064 for urine. In addition, the correlation of nickel levels between each biological sample was low. It was also found that age, gender, commuting time, and different matrices affect concentrations. Blood and serum nickel levels were high in this study compared to other nationwide data, with urinary nickel ranking the second highest among the six countries examined. Therefore, biomonitoring study in the general population should be conducted, and finding a suitable matrix that can reflect nickel exposure to set exposure guideline levels is imperative.

**Supplementary Information:**

The online version contains supplementary material available at 10.1007/s11356-023-31252-7.

## Introduction

Nickel is a naturally occurring heavy metal that, similar to lead, mercury, and cadmium, is hazardous as it can cause acute or chronic health problems (Alissa and Ferns 2011; Park 2010). Nickel combined with other elements, such as sulfur, chlorine, and oxygen, to form nickel compounds (ATSDR 2005), has been linked to lung cancer and classified as International Agency for Research on Cancer (IARC) group 1 compounds (Anttila et al. 1998; Kaldor et al. 1986). Furthermore, workers in industries such as plating and refining may be exposed to nickel and contract chronic bronchitis, leading to decreased lung function and an increased mortality rate from lung diseases (Cornell and Landis 1984; Sunderman Jr 1993). Nickel is mainly exposed through consumption of chocolate, coffee, legumes, and contaminated water in the general population. (ATSDR 2005; Haber et al. 2000; Lyon 1994; SeyyediBidgoli et al. 2022). In addition, nickel can be released into the air by dust emissions from factories, coal combustion, waste incineration, and cigarette smoking is also a known source of nickel exposure (Ghaderi et al. 2023; Yang and Ren 2010).

The National Health and Nutrition Examination Survey (NHANES) in the USA, the Canadian Health Measures Survey (CHMS) in Canada, and the German Environmental Survey (GerES) in Germany have monitored exposure to lead, mercury, and cadmium in the population and investigated their effects in health. Although there has not been much *in vivo* monitoring of nickel, it can be measured in blood, urine, and body tissues (Duda-Chodak and Blaszczyk 2008). The CHMS measured blood and urinary nickel levels in two rounds (cycles 1 and 2), whereas the GerES measured urinary nickel. Water-soluble nickel compounds, such as nickel chloride and nickel sulfide, are rapidly excreted in the urine within 1 to 2 days, and the concentration of excreted urinary nickel indicates recent exposure (Angerer and Lehnert 1990; Cronin et al. 1980; Ghezzi et al. 1989). However, other studies have reported nickel levels using various types of samples other than blood and urine, such as hair in the CHMS cycle 5 and breast milk in breastfeeding women in Sweden (Drysdale et al. 2012). Thus, it is necessary to identify biological samples that reliably reflect nickel levels in the body.

In Korea, there are two representative biomonitoring programs — Korea National Health and Nutrition Examination Survey (KNHANES) and the Korea National Environmental Health Survey (KoNEHS). However, monitoring of nickel has not been conducted yet. Due to plating problems in water purifier evaporator, the risk of nickel exposure was highlighted in 2016 (Kim 2016), and related research is still lacking. Therefore, we measured nickel in whole blood, serum, and urine samples in a panel study to determine internal exposure levels. In addition, we also performed repeated measurements over 21 months to calculate seasonal trends and intra- and inter-individual variations in nickel concentrations.

## Materials and methods

### Study subjects

Between January and February 2020, 50 participants were recruited from a university hospital in Busan, South Korea. Each subject agreed to provide 8 mL of blood and 10 mL of urine samples four times: in March and August 2020 and in June and November 2021. Additionally, we collected information about their commute times. A total of 167 blood and urine samples were collected. Whole blood samples were collected in EDTA tubes, and serum samples were collected in separate serum test tubes and centrifuged at 3000 rpm for 10 min. Then, the supernatant was extracted and stored at −70 °C until analysis. Spot urine samples were collected in a urine cup, aliquoted, and stored at −70 °C until analysis. All the participants provided written informed consent, and this study was approved by the Institutional Review Board of Dong-A University Hospital (IRB No. 13-010).

### Chemical analysis

#### Sample preparation and instrumental analysis

Whole blood samples were analyzed using inductively coupled plasma mass spectrometry (ICP-MS; 7700x, Agilent, USA). The dilution solution consisted of 2% methanol, 0.05% Triton X-100 (Sigma Aldrich, USA), and 0.2% nitric acid (Kanto Chemical, Japan), and the calibration standard solution was 10 mg/L multi-element calibration standard (Perkin Elmer). Seven calibration standards of 0.05, 0.1, 0.5, 1, 2, 5, and 10 ppb were prepared and used for the blood addition experiments, and the samples were diluted 1:50 for analysis. The diluent contained 2% 1-butanol, 0.05% EDTA, 0.05% Triton X-100, and 1% NH_4_OH (Sigma-Aldrich). A 10 mg/L multielement calibration standard (Agilent Technologies, USA) was used as the calibration standard solution. Serum and urine samples were analyzed with ICP-MS (Nexion 2000, Perkin Elmer, USA). To calibrate the nickel in serum and urine, seven calibration standards of 0.05, 0.1, 0.5, 1, 5, 10, and 20 ppb were used, and each sample was diluted 1:10 for analysis. Rhodium (10 mg/L; Agilent Technologies) was used as an internal standard for serum and urine analyses. The ICP-MS analysis conditions were as follows: radio frequency power was set to 1600 W; the sampler and skimmer cone were platinum; the nebulizer type was set to a concentric nebulizer to optimize the analysis; and the analysis mass was set to ^60^Ni (Table S1).

#### Quality assurance and quality control (QA/QC)

The analytical quality was validated using the reference material and blanks. Quintuplicates of the two concentrations (low and high) within the expected concentration range were evaluated for precision and accuracy using inter- and intra-batch measurements. For nickel analysis, the reliability of the analysis is ensured using reference materials provided in accordance with the Clinical Sample Preparation Guide from Agilent Technologies (Santa Clara, USA) and PerkinElmer (Massachusetts, USA), and by adjusting the instrument to the sample and laboratory conditions before performing the analysis. ClinChek levels 1 and 2 (RECIPE Chemicals, Germany) were used for blood and serum nickel analyses, and serotonin levels 1 and 2 (Seronorm, Sweden) were used for urine nickel analysis in every batch. The results showed that the accuracy and precision of all three matrices were within ± 15%, complying with the U.S. Food and Drug Administration reference standards for bioanalytical methods (FDA 2001). For blood, the accuracy of the analytical method ranged from 100.1 to 113.8%. The inter-batch precision ranged from 5.1 to 5.3%, and the intra-batch precision ranged from 3.9 to 8.3%. For serum, the accuracy ranged from 93.2 to 114.5%, the inter-batch precision ranged from 5.5 to 14.3%, and the intra-batch precision ranged from 3.1 to 13.5%. For urine, the accuracy ranged from 92.8 to 99.5%; the inter-batch precision ranged from 2.4 to 10.9%; and intra-batch precision ranged from 2.3 to 9.3% (Tables S2 and S3). For external quality control, the reliability of the analytical method used for the annual analysis of heavy metals was verified using the German External Quality Assessment Scheme (G-EQUAS). Depending on the time of collection, each analysis had a different limit of detection (*LOD*) value. In 2022, the *LOD* for blood was 0.033 g/L. The LOD for serum was 0.056 μg/L in 2020 and 0.091 μg/L in 2021. The *LOD* in urine was 0.226 μg/L in 2020 and 0.253 μg/L in 2021.

### Statistical analysis

One urine sample collected in August 2020 with a value below the *LOD* was included in the analysis by dividing the measured value by 2 (Yoon 2005). In addition, one out of 50 blood samples collected in March 2020 had an abnormal analytical value and was excluded from the statistical analysis. To ensure the normality of the nickel concentrations, statistical analysis was performed after natural logarithmic transformation. Geometric means and 95% confidence intervals (*CI*s) calculated for survey season, gender, age, and commute time. Secondly, one-way analysis of variance (ANOVA) was performed to test for differences in seasonal means, followed by Bonferroni’s post hoc comparison analysis. Correlation analysis was performed to analyze associations between matrices. Next, we conducted multiple regression analyses for blood, serum, and urinary nickel to determine the effects of gender, age, commute time, and other matrices as well seasonal variation. In addition, linear mixed model was used to consider the values of each sampling time. Final model includes sampling season, gender, age, commute time, interaction between age and commute time, and the effects of other matrices as a fixed effect, and the variation of nickel concentration of each participant as a random effect. The reference group for the linear mixed model and multiple regression analysis was set to August 2020 as the sampling time, and gender was set to female. In addition, within- and between-subject variations were determined using a linear mixed model with a restricted maximum likelihood (REML) method. The fixed-effects term was designated as the sampling period (seasonal effects), and the covariance structure was set to the compound symmetry type. The ratio of inter-individual variability to total subject variability (inter-class correlation; *ICC*) was calculated using the covariance parameter estimate and residual variance. For the final step, the concentration of fine particulate matter in the air was analyzed. We used the data from Air Korea (www.airkorea.or.kr ), which is a national air quality monitoring system organized by the Korea Environment Corporation (K-eco). The raw data provided hourly arithmetic averages from 00:00 to 24:00 for the sampling years from 2020 to 2021. Particulate matter — PM_10_ and PM_2.5_ were categorized into spring (March to May), summer (June to August), autumn (September to November), and winter (December to February). Geometric means and 95% *CI*s were calculated to identify seasonal trends in PM concentrations. Statistical analyses were performed at a significance level (alpha) of less than 5%, 10% using SAS 9.4 (SAS Institute, Cary, NC, USA).

## Results

### Nickel distribution in the study population

Of the 50 participants that participated in the first round, 48 participated in the second round, 35 in the third round, and 34 in the final round in November 2021, resulting in 68% of all participants being followed up. As of March 2020, the average age of all participants was 36.5 years, with 36.6 years for men and 36.5 years for women (range: 20–55 years). To explore the distribution of nickel levels across the samples, we presented nickel levels and percentiles according to age and sex. The overall geometric means were 1.028 μg/L in blood, 0.687 μg/L in serum, and 1.464 μg/L in urine. The concentrations of biological samples according to sex were 0.980 μg/L for men and 1.074 μg/L for women in blood, 0.676 μg/L for men and 0.697 for women in serum, and 1.401 μg/L for men and 1.527 for women in urine. Age-specific concentrations in all three biological samples were the highest in those aged 40–49 years (blood: 1.076 μg/L, serum: 0.760 μg/L, and urine: 1.669 μg/L). Nickel concentrations in blood were the highest at 1.018 μg/L in the 120 min to the highest commute category, while serum was the highest in the 91 to 120 min category, and urine was the highest in the 31 to 60 min category (serum: 0.713 μg/L and urine: 1.578 μg/L) (Table 1).

**Table 1:** Nickel distribution in the body by gender, age and commute time (unit: μg/L)

Matrix	Blood_Ni		Serum_Ni		Urine_Ni	
Variable	Sample (%)	GM (95% *CI*)	Sample (%)	GM (95% *CI*)	Sample (%)	GM (95% *CI*)
Total	166	1.028 (0.978, 1.081)	167	0.687 (0.642,0.735)	167	1.464 (1.335, 1.607)
Sex						
Male	80 (48.2)	0.980 (0.906, 1.060)	81 (48.5)	0.676 (0.606, 0.754)	81 (48.5)	1.401 (1.221, 1.607)
Female	86 (51.8)	1.074 (1.008, 1.145)	86 (51.5)	0.697 (0.641, 0.758)	86 (51.5)	1.527 (1.344, 1.734)
Age						
19–29	55 (33.1)	1.019 (0.945, 1.124)	57 (34.1)	0.648 (0.572, 0.741)	57 (34.1)	1.356 (1.160, 1.572)
30–39	57 (34.3)	1.065 (0.990, 1.156)	56 (33.5)	0.695 (0.615, 0.774)	56 (33.5)	1.470 (1.296, 1.663)
40–49	28 (16.9)	1.076 (0.952, 1.212)	28 (16.8)	0.760 (0.668, 0.908)	28 (16.8)	1.669 (1.242, 2.206)
50–59	26 (15.7)	0.918 (0.828, 1.038)	26 (15.6)	0.682 (0.553, 0.840)	26 (15.6)	1.494 (1.210, 1.873)
Commute time						
0–30 min	54 (32.5)	1.066 (1.153, 0.986)	55 (32.9)	0.68 (0.778, 0.594)	55 (32.9)	1.366 (1.594, 1.17)
31–60 min	57 (34.3)	1.017 (1.124, 0.920)	57 (34.1)	0.704 (0.789, 0.628)	57 (34.1)	1.578 (1.861, 1.338)
61–90 min	23 (13.9)	0.996 (1.166, 0.851)	23 (13.8)	0.660 (0.802, 0.543)	23 (13.8)	1.509 (1.931, 1.179)
1–120 min	24 (14.5)	1.003 (1.108, 0.908)	24 (14.4)	0.713 (0.845, 0.601)	24 (14.4)	1.381 (1.730, 1.103)
Over 120 min	8 (4.8)	1.018 (1.379, 0.751)	8 (4.8)	0.600 (0.731, 0.493)	8 (4.8)	1.503 (3.557, 0.635)

*Ni* nickel, *GM* geometric mean, *CI* confidence interval

### Seasonal changes in nickel levels

**Fig. 1:**
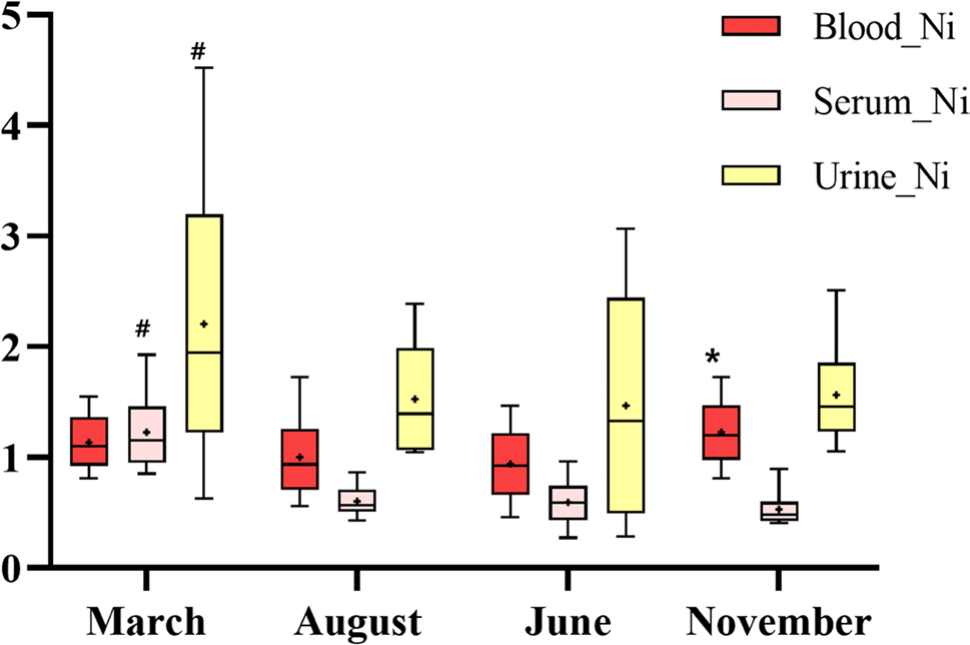
The distribution of Nickel by sampling season. * Nickel in blood is significantly higher in November compared to June and August; # Serum and urinary nickel significantly higher in March compared to other months (one-way ANOVA with Bonferroni test, *p value* < 0.05)

As shown in fig. 1, the geometric mean of blood nickel was the highest in November at 1.197 μg/L (95% *CI*: 1.109, 1.292) (*p*-value: 0.004), followed by March, at 1.116 μg/L; June, at 0.928 μg/L; and August, at 0.914 μg/L. The geometric mean of serum nickel was the highest in March 2020, at 1.146 μg/L (95% *CI*: 1.068, 1.231), almost twice as high as that in June, August, and November (*p*-value: < 0.001). The geometric mean of nickel in urine was also the highest in March 2020, at 1.893 μg/L (95% *CI*: 1.608, 2.229) (*p*-value: 0.001).

### Association of biological samples and intra-individual variations

**Fig. 2:**
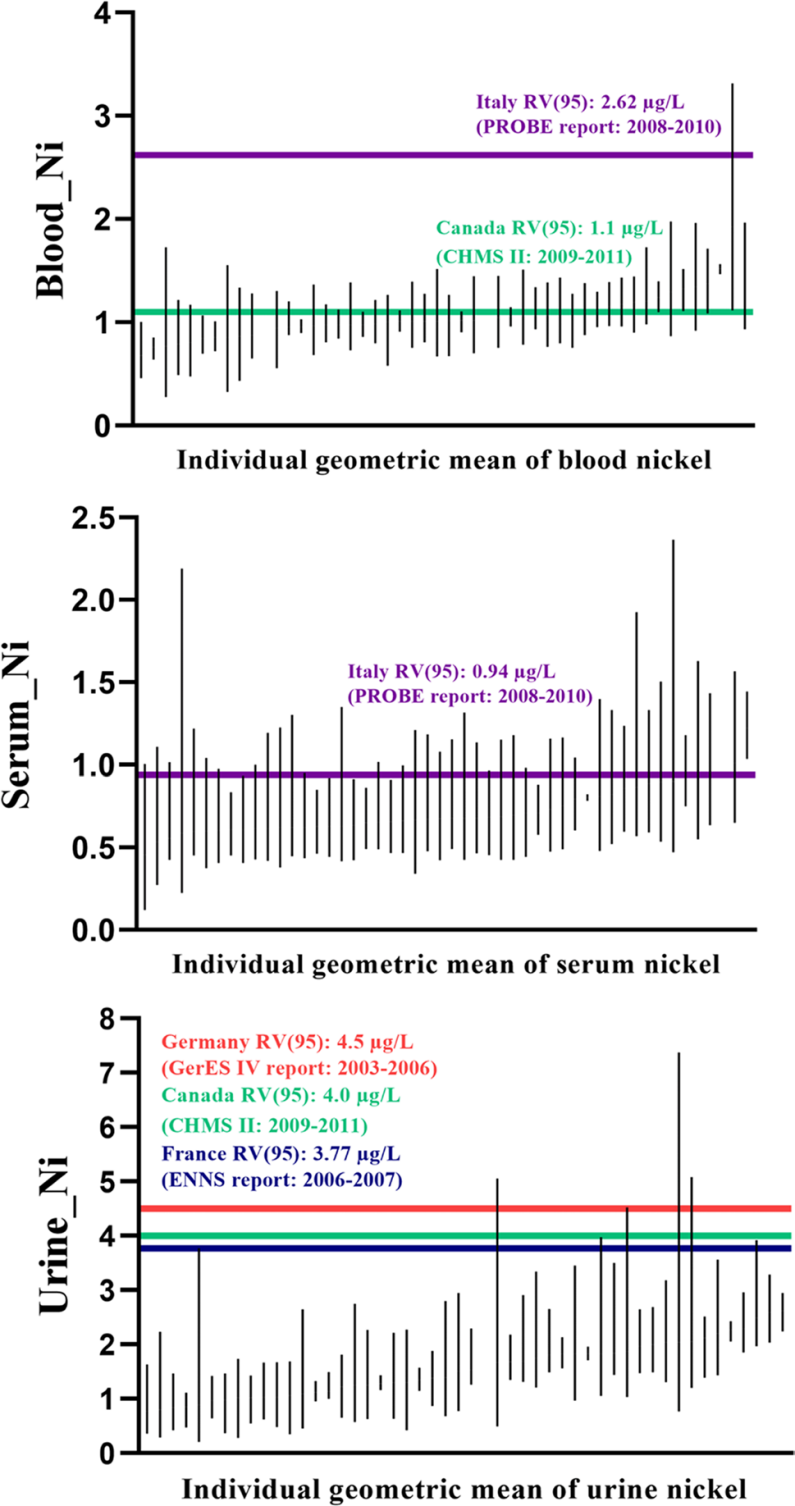
Trends of variability of the repeated measurements for nickel — blood (top), serum (middle), and urine (bottom). Vertical lines indicate the range of individual nickel concentrations

Correlations between biological samples were as follows: *ρ* = 0.10 for blood and serum nickel, *ρ* = 0.30 for urinary and serum nickel, and *ρ* = 0.18 for blood and urinary nickel, all of which were rather low (Figure S1). Figure 2 showed the within individual variation. Each vertical line presents the range of the lowest and the highest concentrations within individuals. Inter-individual correlations were 0.081 for blood nickel and 0.064 for urinary nickel. *ICC*s could not be calculated for serum because the degree of intra-individual variation was very large compared with the inter-individual variation.

### Multiple regression and mixed model

In the result of regression (Table 2), seasonal effect of November 2021 was significantly higher than those of August 2020 (*p*-value: < 0.0001). Commute time and the interaction of age and commute time also showed a significant difference in blood nickel concentration (*p*-value: 0.02 and 0.022, respectively). For serum nickel, the seasonal effect of March 2020 was significant (*p*-value: < 0.0001), and the November 2021 concentration was 0.187 μg/L lower than the August 2020 concentration (*p*-value: 0.009). In the case of urinary nickel, the seasonal effects of March 2020, June 2021, and November 2021 had been significantly higher than the reference group of August 2020. The effects of age, commute time, and the interaction of age and commute time were significant concentrations (*p*-value: 0.007, 0.012, and 0.016, respectively).

**Table 2:** Multiple regression and mixed effect model for blood, serum, urine nickel

Blood	Regression		Mixed model		Serum	Regression		Mixed model		Urine	Regression		Mixed model	
	Est	*p value*	Est	*p value*		Est	*p value*	Est	*p value*		Est	*p value*	Est	*p value*
Season: Mar 2020	0.137	0.176	0.126	0.176		0.636	<.0001	0.624	<.0001		0.494	0.003	0.568	0.001
Season: Jun 2021	0.030	0.704	0.027	0.704		0.031	0.643	0.009	0.893		0.371	0.004	0.371	0.005
Season: Nov 2021	0.338	<.0001	0.318	<.0001		−0.187	0.009	−0.216	0.003		0.334	0.016	0.315	0.034
Sex: male	−0.055	0.323	−0.109	0.033		−0.046	0.321	−0.011	0.819		−0.062	0.497	-0.043	0.687
Age	0.007	0.215	0.004	0.454		0.007	0.160	0.005	0.248		0.026	0.007	0.019	0.074
Commute time	0.006	0.020	0.003	0.278		0.001	0.749	0.002	0.492		0.011	0.012	0.009	0.065
Age × commute time	0.000	0.022	−0.0001	0.167		−0.00004	0.500	−0.0001	0.411		−0.0003	0.016	−0.0002	0.079
Serum_Ni	0.216	0.027	0.154	0.103	Blood_Ni	0.153	0.027	0.127	0.103	Blood_Ni	0.071	0.599	0.097	0.557
Urine_Ni	0.026	0.599	0.030	0.503	Urine_Ni	0.028	0.516	0.012	0.766	Serum_Ni	0.104	0.516	0.0004	0.998

The reference group for time of sampling is August 2020, and the reference group for gender is female. *p value* < 0.05

*Mar* March, *Jun* June, *Nov* November, *Ni* nickel, *Est* estimate

The results of the mixed model considering individual repetition effect were consistent with the results of the multiple regression analysis, with no significant effect of other variables. Blood nickel was 0.318 μg/L higher in November 2021 than in August 2020 (*p*-value: < 0.0001). For serum nickel, the March 2020 concentration was 0.624 μg/L higher than the August 2020 concentration (*p*-value: < 0.0001), and the November 2021 concentration was 0.216 μg/L lower than the August 2020 concentration (*p*-value: 0.003). The effects of age, commute time, and the interaction of age and commute time had marginal significance on urinary nickel levels.

### Comparison with data from other countries

Compared to other nationwide biomonitoring data, 28% of South Korean individuals had higher blood nickel than the individuals surveyed for the Canadian CHMS cycle 2 (2009–2011) (reference value, *RV*95: 1.1 μg/L), and 12% of South Korean subjects had higher serum nickel than Italian individuals surveyed for the PROBE report (2008–2010) (*RV*95: 0.94 μg/L) (Fréry et al. 2017; Haines et al. 2017; Hmsc 2013). The horizontal lines in Fig. 2 indicated the international reference values. Table 3 shows that South Korean individuals had lower levels compared to the international *RV*95s for urinary nickel such as the Canadian CHMS Cycle 2 (*RV*95: 4.0 μg/L), German GerES IV report (2003–2006) (*RV*95: 4.5 μg/L), Belgian study (2010–2011) (*RV*95: 4.73 μg/L), and the French ENNS report (2006–2007) (*RV*95: 3.77 μg/L) (Choi et al. 2017; Hmsc 2013; Hoet et al. 2013; Schulz et al. 2009).

**Table 3:** Reference values for nickel concentration by country

Country	Matrix	*GM*	(95% *CI*)	*RV*95	(95% *CI*)	Reference population	Study period	Reference
Korea	Blood	1.03	(0.98, 1.08)	N/A	N/A	Korean population (20–55)	2020–2021	This study
	Serum	0.69	(0.64, 0.74)					
	Urine	1.46	(1.34, 1.61)					
Canada (cycle 1)	Blood	0.63	(0.57, 0.70)	1.6	(1.32, 2.08)	Canadian population (age 6–79)	2007–2009	CHMS I report (Haines et al. 2017; Hmsc 2013)
	Urine	1.3	(1.30, 1.40)	4.5	(3.90, 5.00)			
Canada (cycle 2)	Blood	0.48	(0.45, 0.51)	1.1	(1.10, 1.20)	Canadian population (age 3–79)	2009–2011	CHMS II report (Haines et al. 2017; Hmsc 2013)
	Urine	1.2	(1.20, 1.30)	4.0	(3.50, 4.40)			
Germany	Urine	1.26	N/A	4.50	(4.20, 4.72)	Children (age 3–14)	2003–2006	GerES IV report (Schulz et al. 2009, 2011)
Belgium	Urine	1.73	(1.64, 1.83)	4.73	(4.55, 5.23)	Belgium adults (age > 18)	2010–2011	Hoet et al. (2013)
France	Urine	1.23	(1.17, 1.28)	3.77	(3.49, 3.97)	French adults (age 18–74)	2006–2007	ENNS report (metals) (Fréry et al. 2017)
Italy	Blood	0.89	(0.86, 0.92)	2.62	N/A	Adults (age 18–65)	2008–2010	PROBE report (Choi et al. 2017; Schulz et al. 2009)
	Serum	0.35	(0.34, 0.37)	0.94				

*GM* geometric mean, *CI* confidence interval, *RV*95 reference value

## Discussion

Despite the potential toxicity of nickel, few exposure assessment studies have been conducted to date. Therefore, we repeatedly measured nickel concentrations in whole blood, serum, and urine samples to determine the distribution of nickel concentrations in the body and seasonal effects. The study was conducted in Busan which is the southern part of South Korea, which is characterized by four distinct seasons (spring: March to May; summer: June to August; autumn: September to November; and winter: December to February), and the average annual temperature is 15.0 °C. This study analyzed the concentration of nickel in the body in each season except winter. The highest blood nickel levels were in November and March, and the highest serum and urine nickel levels were in March. These results can be attributed to the influence of yellow dust and fine particulate matter that usually occur in the spring (Korea Ministry of Environment 2019). In our study, we analyzed official air quality data (Air Korea, Figure S2) and found that PM_10_ was highest in March–May (2020: 29.98 μg/m^3^ and 2021: 32.10 μg/m^3^) in both study years. PM_2.5_ was highest in December–February (2020: 17.50 μg/m^3^ and 2021: 16 μg/m^3^). PM_10_ and PM_2.5_ showed similar trends in both 2020 and 2021, with the lowest concentrations in June–August in both years (PM_10_: 21.08 μg/m^3^ (2020) and 18.73 μg/m^3^ (2021); PM_2.5_: 11.20 μg/m^3^ (2020) and 9.37 μg/m^3^ (2021)). Participants in the study showed similar trends to the 2020 and 2021 fine particulate matter concentrations in South Korea, with higher levels of nickel in November and March compared to June and August.

Another study in South Korea found that trace heavy metals in the air increase by an average of 5.3% per year, and that Busan has the highest concentrations of chromium, nickel, and iron in the air among the country’s seven largest cities (Korea Institute of Health and Environment, 2022). Transition metals such as nickel were found to be mainly distributed in PM_2.5_ and PM_10_ (Shaheen et al. 2005). In addition, PM_2.5_ has been found to contain more nickel, lead, cadmium, and vanadium than other heavy metals (Espinosa et al. 2001). This is a concern as nickel in airborne ultrafine particulate matter can enter the body through the respiratory tract. Larger particles are mainly deposited in the nasopharyngeal region, while smaller particles can be adsorbed by the bronchi and alveoli and accumulate in the body (Klaassen 2013). Therefore, it is likely that nickel contained in fine particulate matter enters the body through the respiratory tract during the winter to spring season, resulting in high body nickel concentrations. Interestingly, our results showed that commuting time is associated with nickel concentration. It is likely to expose to particulate matter more as increasing the commuting time (Table 2). And it brings to increase the nickel concentration in human body. Since the composition ratio of heavy metals in fine dust was not performed; however, it is limited to explain nickel in the air as the cause of the association between commuting time and nickel concentrations in the human body.

The geometric mean for blood nickel in this study was 1.03 μg/L, which was higher than the Canadian geometric mean for cycles 1 and 2. In addition, 4% and 28% of the 50 participants of our study had blood nickel values higher than the Canadian *RV*95 values in cycles 1 and 2, respectively (Saravanabhavan et al. 2017). The blood geometric mean in this study was higher than that from the Italian PROBE (2008–2010) study; still, no individual had a geometric mean higher than their *RV*95 value. The geometric mean for serum nickel in our study was about twice that of the Italian PROBE study. Approximately 12% of the individuals in our study had serum nickel levels higher than the *RV*95 from the Italian study. Both in this study and the Italian study, blood nickel was higher than serum nickel (Choi et al. 2017; Haines et al. 2017). The geometric mean for nickel in urine in this study was lower than that in the Belgian study, but higher than that in the Canadian CHMS (cycles 1 and 2), German, and French studies. None of the study participants had a measurement higher than the *RV*95 from the Canadian, German, Belgian, or French studies. However, our study had methodical differences from the others. Here, we analyzed all biological samples using ICP-MS, whereas the Italian study used sector field inductively coupled plasma mass spectrometry (SF-ICP-MS) to analyze blood and serum; the German study was conducted in children and adolescents (3–14 years old); and most international studies took samples in the morning after an overnight fast (Fréry et al. 2017; Nielsen et al. 1999; Saravanabhavan et al. 2017; Schulz et al. 2009, 2011; Waseem and Arshad 2016). Therefore, direct comparisons of our results should be made with caution as there may be differences in body nickel concentrations that depend on the analytical equipment, dietary restrictions, and characteristics of each individual. Moreover, this study is not very representative due to the small sample size.

The greatest distribution of nickel in postmortem human tissues is in the lungs, thyroid, and adrenal glands (Dunnick et al. 1989; Rezuke et al. 1987). Orally absorbed nickel is generally distributed in the kidneys (EFSA 2015). Peak blood nickel concentrations occur within 1.5 to 3 h of oral exposure, with an average half-life of 60 h (Hendel and Sunderman Jr 1972). The half-life of serum nickel, 11 to 39 h have been reported for water-soluble nickel and 30 to 54 h for non-water-soluble nickel (Sunderman Jr 1989; Sunderman Jr. et al. 1984). Nickel sulfate, a water-soluble form of nickel, has been reported to be absorbed about 40 times more easily and causing a sharp rise in blood nickel levels ingested with drinking water than with food. Non-water-soluble nickel compounds such as nickel oxide and nickel carbonate accumulate in the respiratory tract and lungs and gradually release nickel into the bloodstream (Mahiya et al. 2014; Sunderman Jr 1989; Torjussen and Andersen 1979). Then, the absorbed nickel is excreted in the urine (Ghezzi et al. 1989; Patriarca et al. 1997).

In this study, nickel in blood, serum, and urine were measured repeatedly, and the *ICC* was calculated from the three biological samples. The calculated *ICC*s showed a large intra-individual variation in body nickel concentrations and a low correlation between biological samples. Since dietary restrictions were not considered in this study, it is possible that the source, timing of exposure, and urinary excretion of nickel may have influenced body nickel levels in different individuals. Therefore, major sources and routes of exposure should be considered before selecting samples for collection in nickel exposure studies.

The link between nickel and carcinogenicity was first reported by several epidemiological studies, including Auler, who reported 47 nasal cancer diagnoses and 82 lung cancer diagnoses among nickel smelter workers, and animal studies (Auler and Adam 1937; Kasprzak et al. 2003; Sunderman 1968). Epidemiological studies have shown that chronic exposure to fumes from nickel smelting is associated with increased mortality from malignant lung and nasal cavity tumors (Denkhaus and Salnikow 2002). Among animal studies related to nickel carcinogenicity, Campbell (1943) reported a twofold increase in the incidence of lung cancer in rats chronically exposed to nickel (J. A. Campbell 1943), and another study found kidney tumors in rats that received nickel sulfide, which was shown to increase erythropoietin levels and increase red blood cell levels (Sunderman Jr et al. 1982; Sunderman Jr. et al. 1984). In addition, when 17 nickel compounds were given to different groups of rats, nine groups developed kidney cancer within 2 years (Sunderman Jr. et al. 1984). These studies show that chronic exposure to nickel can affect the lungs, nasal passages, and kidneys. Therefore, thresholds for nickel concentrations in the body should be established, and appropriate biomarkers are needed to monitor them and ensure public health.

Our study results showed that the intra-individual variability of nickel is very high, which could be influenced by an individual’s lifestyle, eating habits, and physical condition at the time of the survey. The limitation of this study is that we did not consider these influential factors. Additionally, the sample size is small and insufficient to represent the general population. Given the paucity of research on nickel, however, this study is significant as it used three types of samples (blood, serum, and urine) to determine individual nickel levels according to seasonal measurements and compared them to international equivalents. Another major strength of this study is that the *ICC* values of nickel in the three biological matrix were calculated using four repeated measurements. Furthermore, based on multiple regression and mixed-effects model analysis, we found that urine may be the most sensitive of the three biomarkers. In future studies, detailed biomonitoring with 24-h urine sampling will be required, and validation studies in larger populations are needed to analyze the relationship between nickel concentrations and health effects.

## Conclusion

This study showed that nickel concentrations varied with sampling time, with higher concentrations in March and November, which matched the seasonal trends for fine particulate matter concentrations from 2020 to 2021. In addition, the correlation between the biological matrices of nickel was low. We found greater intra-individual variation than inter-individual variation, suggesting that nickel levels in the body may not be stable enough to be used as a biomarker. The inter-individual correlation was low, most likely because of the nature of nickel, which has large concentration differences among samples depending on the route of exposure, and the lack of consideration for dietary restrictions. However, multiple regression and mixed-model analyses showed seasonal variation in all three biological samples. In particular, urinary nickel was affected by age, commute time, and duration of interaction. Though further research is needed, this study suggests that urine would be a relatively sensitive indicator of nickel exposure. Future studies should be conducted to determine nickel exposure levels in the general population and to establish health effect thresholds for nickel.

### Supplementary information

ESM 1 (DOCX 239 kb)

## Abbreviations

ANOVA: Analysis of variance
CHMS: Canadian Health Measures Survey
*CI*: Confidence interval
GerES: German Environmental Survey
IARC: International Agency for Research on Cancer
*ICC*: Inter-class correlation
ICP-MS: Inductively coupled plasma mass spectrometry
KNHANES: Korea National Health and Nutrition Examination Survey
KoNEHS: Korean National Environmental Health Survey
*LOD*: Limit of detection
NHANES: National Health and Nutrition Examination Survey
QA/QC: Quality assurance and quality control
*REML*: Restricted maximum likelihood
*RV*: Reference value
