# Supplementary material for: Characterization of nickel levels considering seasonal and intra-individual variation using three biological matrices
Source: Environ Sci Pollut Res Int. 2023 Dec 8;31(2):2546–54. doi: 10.1007/s11356-023-31252-7 (PMC10791924; doi:10.1007/s11356-023-31252-7)
Supplement: Supplementary file 1 — (DOCX 239 kb) [file 11356_2023_31252_MOESM1_ESM.docx]

List of Supplementary Table

[Table S1. ICP-MS set up for nickel measurement](#_Toc131714044)

[Table S2. Inter-batch precision and accuracy of quality control sample data](#_Toc131714045)

[Table S3. Intra-batch precision and accuracy of quality control sample data](#_Toc131714046)

Figure S1. Correlations of nickel concentrations between the biological samples

Figure S2. Seasonal Fine Dust Concentration (2020~2021)

| Table S1. ICP-MS set up for nickel measurement | | |  |  |
| --- | --- | --- | --- | --- |
| **Perkin Elmer Nexion 2000** | | | **Agilent 7700x** | |
| **Parameter** | **Matrix** | | **Parameter** | **Matrix** |
|  | **Serum** | **Urine** |  | **Blood** |
| **Radio Frequency Power** | 1600 W | 1600 W | **Radio Frequency Power** | 1600 W |
| **Carrier Gas** | 0.6 L/min | 1.95 L/min | **Nebulizer gas flow** | 0.8∼1.05 (optimized daily) |
| **Sample Depth** | 6.0 mm | 8.0 mm | **Plasma gas flow** | 15 L/min |
| **Sampler and skimmer cones** | Platinum | Platinum | **Auxiliary gas flow** | 1.2 L/min |
| **Spray chamber Temperature** | 2℃ | 2℃ | **Sampler and skimmer cones** | Platinum |
| **Nebulizer type** | Concentric nebulizer | Concentric nebulizer | **Spray chamber** | Cyclonic |
| **Nebulizer pump uptake rate** | 0.1 rps | 0.1 rps | **Nebulizer type** | Concentric nebulizer |
| **The flow Rate** | 5.5 mL/min | 4.5 mL/min | **Scan mode** | Peak Hoping |
| **Analytical masses** | ^60^Ni | ^60^Ni | **Analytical masses** | ^60^Ni |

| Table S2. Inter-batch precision and accuracy of quality control sample data | | | | | | | (Unit: µg/L) |
| --- | --- | --- | --- | --- | --- | --- | --- |
| **Matrix** | **Year** | **n** | **Reference value** | | **Measured** | **Precision (CV %)** | **Accuracy (%)** |
| **Blood** | 2022 | 5 | Low | 2.10 | 2.39 ± 0.11 | 5.111 | 113.7 |
|  |  |  | High | 4.53 | 4.53 ± 0.24 | 5.253 | 100.1 |
| **Serum** | 2020 | 5 | Low | 5.46 | 5.37 ± 0.55 | 9.974 | 98.35 |
|  |  |  | High | 9.00 | 9.79 ± 0.58 | 6.472 | 108.7 |
|  | 2021 | 4 | Low | 2.05 | 2.34 ± 0.11 | 5.476 | 114.1 |
|  |  |  | High | 5.59 | 5.24 ± 0.80 | 14.25 | 93.78 |
| **Urine** | 2020 | 4 | Low | 3.24 | 3.22 ± 0.35 | 10.86 | 99.46 |
|  |  |  | High | 40.70 | 39.4 ± 1.79 | 4.403 | 96.76 |
|  | 2021 | 4 | Low | 3.24 | 3.08 ± 0.23 | 7.194 | 95.06 |
|  |  |  | High | 14.60 | 14.2 ± 0.35 | 2.387 | 97.59 |

| Table S3. Intra-batch precision and accuracy of quality control sample data | | | | | | | (Unit: µg/L) |
| --- | --- | --- | --- | --- | --- | --- | --- |
| **Matrix** | **Year** | **days** | **Reference value** | | **Measured** | **Precision (CV %)** | **Accuracy (%)** |
| **Blood** | 2022 | 5 | Low | 2.10 | 2.39 ± 0.17 | 8.288 | 113.8 |
|  |  |  | High | 4.53 | 4.65 ± 0.18 | 3.902 | 102.6 |
| **Serum** | 2020 | 5 | Low | 5.46 | 5.46 ± 0.51 | 9.119 | 96.87 |
|  |  |  | High | 9.00 | 9.75 ± 0.82 | 9.153 | 108.3 |
|  | 2021 | 4 | Low | 2.05 | 2.35 ± 0.06 | 3.134 | 114.5 |
|  |  |  | High | 5.59 | 5.21 ± 0.76 | 13.52 | 93.20 |
| **Urine** | 2020 | 4 | Low | 3.24 | 3.07 ± 0.30 | 9.324 | 94.83 |
|  |  |  | High | 40.70 | 38.5 ± 1.49 | 3.656 | 94.01 |
|  | 2021 | 4 | Low | 3.24 | 3.01 ± 0.27 | 8.182 | 92.82 |
|  |  |  | High | 14.60 | 14.3 ± 0.34 | 2.343 | 98.04 |


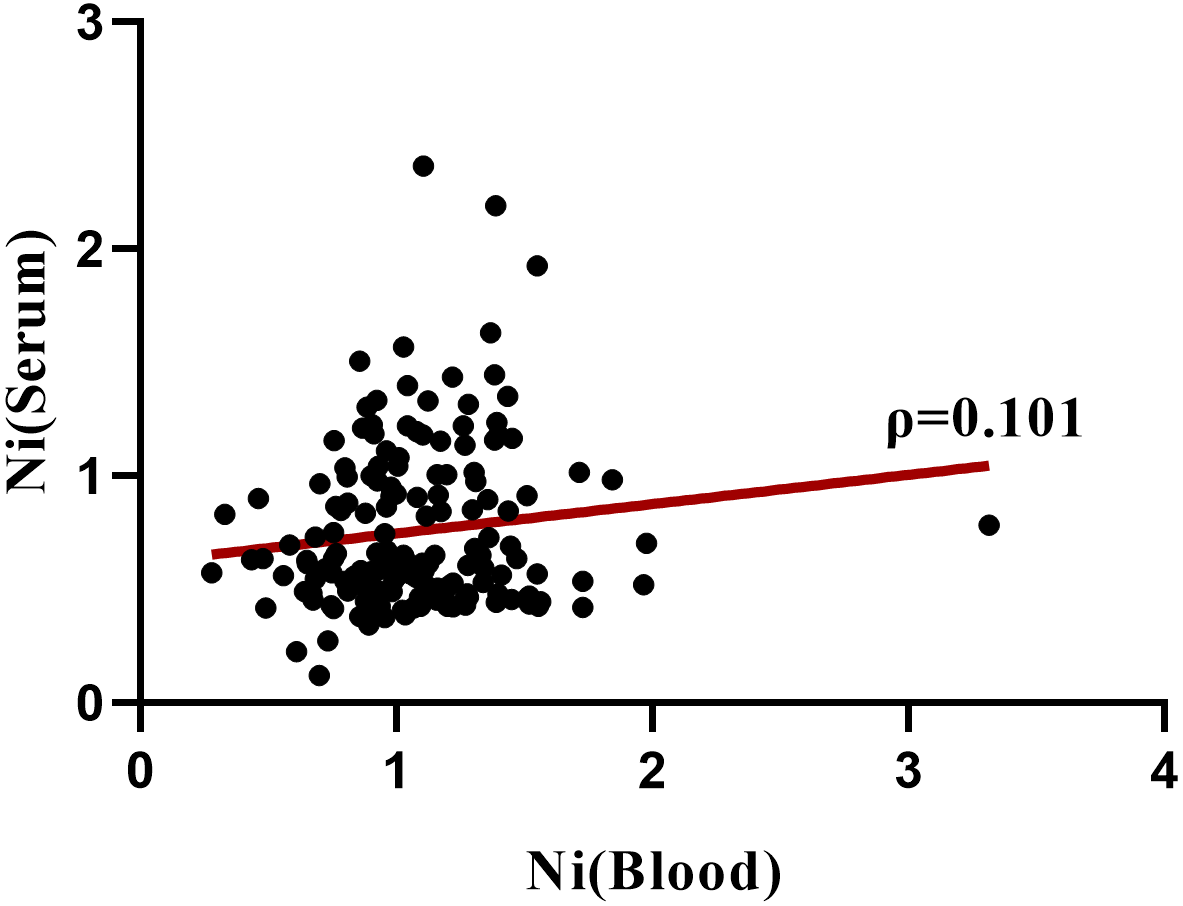


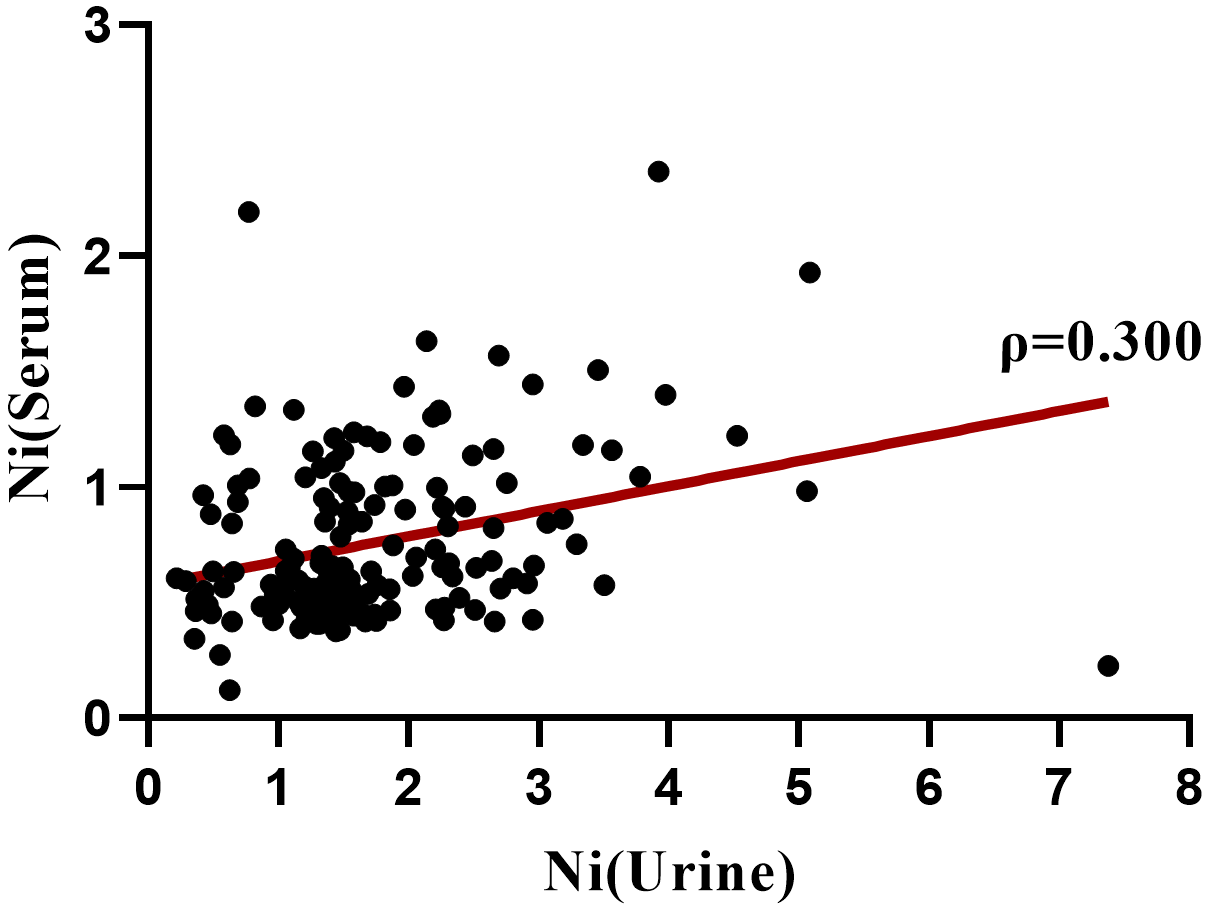


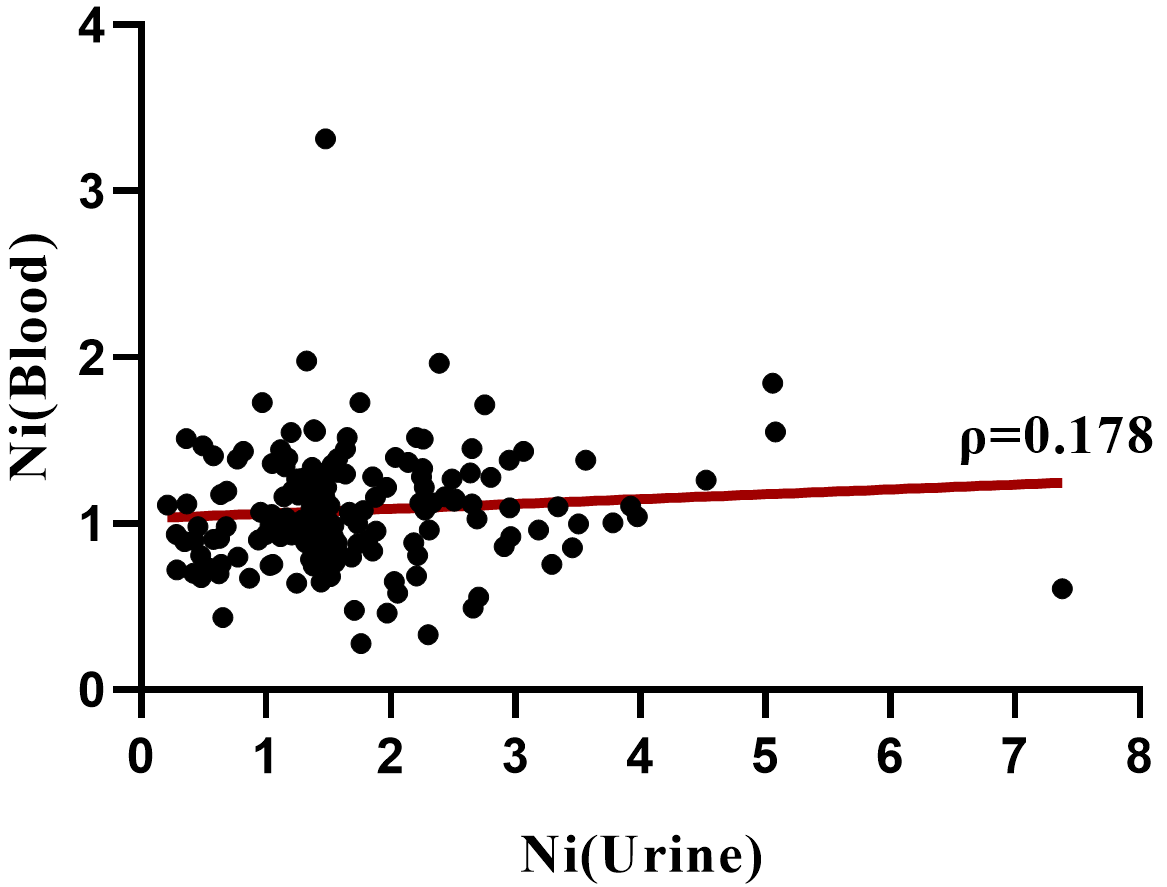


Figure S1. Correlations of nickel concentrations between the biological samples

Figure S2. Seasonal Fine Dust Concentration (2020~2021) (Unit: µg /m³)
